# RAF-1 Mutation Associated with a Risk for Ventricular Arrhythmias in a Child with Noonan Syndrome and Cardiovascular Pathology

**DOI:** 10.2478/jccm-2022-0007

**Published:** 2022-05-12

**Authors:** Amalia Făgărășan, Hamida Al Hussein, Simina Elena Ghiragosian Rusu

**Affiliations:** 1 George Emil Palade University of Medicine, Pharmacy, Science, and Technology of Targu Mures, Targu Mures Romania

**Keywords:** Noonan syndrome, child, tachyarrhythmia, RAF-1 mutation

## Abstract

**Introduction:**

Noonan syndrome (NS) is a dominant autosomal disease, caused by mutations in genes involved in cell differentiation, growth and senescence, one of them being RAF1 mutation. Congenital heart disease may influence the prognosis of the disease.

**Case presentation:**

We report a case of an 18 month-old female patient who presented to our institute at the age of 2 months when she was diagnosed with obstructive hypertrophic cardiomyopathy, pulmonary infundibular and pulmonary valve stenosis, a small atrial septal defect and extrasystolic arrhythmia. She was born from healthy parents, a non-consanguineous marriage. Due to suggestive phenotype for NS molecular genetic testing for RASopathies was performed in a center abroad, establishing the presence of RAF-1 mutation. Following rapid progression of cardiac abnormalities, the surgical correction was performed at 14 months of age. In the early postoperative period, the patient developed episodes of sustained ventricular tachycardia with hemodynamic instability, for which associated treatment was instituted with successful conversion to sinus rhythm. At 3-month follow-up, the patient was hemodynamically stable in sinus rhythm.

**Conclusions:**

The presented case report certifies the importance of recognizing the genetic mutation in patients with NS, which allows predicting the severity of cardiac abnormalities and therefore establishing a proper therapeutic management of these patients.

## Introduction

Noonan syndrome (NS) is a dominant autosomal disease with an incidence of 1 / 1000-2500 newborns for the severe phenotype. It is associated with multiple congenital anomalies (the most common being cardiac abnormalities). Literature data does not show a predilection for race or gender (1,2,3). The high variability of clinical manifestations makes it difficult to diagnose patients with mild forms. NS is caused by mutations in genes involved in cell differentiation, growth and senescence, half of which are mutations in the PTPN 11 gene, which is located on the long arm of chromosome 12 and is associated with pulmonary stenosis (PS) and atrial septal defect (ASD) (1,4,5). Other mutations involved are those of SOS 1 gene (13%), RAF1 gene (317%) as well as KRAS gene (5% of cases). Currently, molecular genetic testing can confirm up to 70% of cases (5).

Based on genetic diagnosis, genetic counseling, individualized management as well as a proper prognosis and recurrence risk prediction can be achieved (1,6). Antenatal diagnosis may be considered for fetuses with normal karyotype and increased nuchal translucency, especially when cardiac anomalies or polyhydramnios and/or hydrothorax are present (1,7). Associated congenital heart disease may influence the prognosis of the disease, the most common being PS, hypertrophic cardiomyopathy (HCM) and ASD. However, the combination of these three cardiac malformations is less common, being described in only 5% of the cases (8). Early onset of clinical heart failure in children diagnosed with HCM is associated with a poor prognosis and increased mortality (9).

## Case Report

### Presenting symptoms

We report a case of an 18-month-old female patient who presented to our institute at the age of 2 months when she was admitted due to cyanotic extremities, fatigue while feeding and the presence of a precordial murmur since the neonatal period. She was born from healthy parents, a non-consanguineous marriage, with no history of exposure to teratogens during the antenatal period. No family history of genetic abnormalities or congenital cardiovascular malformations were revealed. The patient was the fourth-born child with a monitored, full-term pregnancy and vaginal delivery, weighing 2900 g, with an Apgar score of 8 at 1 minute postpartum and breastfed exclusively for the first year. The patient was hemodynamically stable at the first admission.

### Diagnostic criteria

**Fig. 1:**
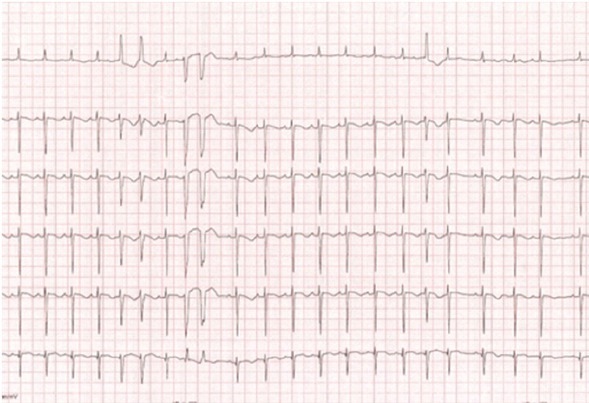
Electrocardiogram: heart rate: 90-120 beats/minute, extrasystolic arrhythmia with supraventricular and ventricular extrasystoles

Physical examination revealed growth restriction, grade 1 malnutrition and suggestive phenotype for NS (facial dysmorphism with lower insertion of the ears, ocular hypertelorism, simian line and microcephaly); cardiovascular examination revealed arrhythmic heart sounds, heart rate of 88-120 beats/min, grade III/6 systolic murmur at the left sternal border, blood pressure of 80/60 mmHg, oxygen saturation of 97-98% and mild hepatomegaly. Electrocardiogram (ECG) at that moment showed extrasystolic arrhythmia with polymorphic supraventricular and ventricular extrasystoles (Fig.1).

**Fig. 2:**
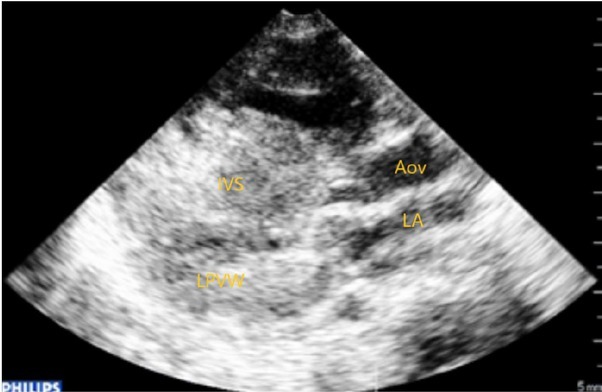
Transthoracic echocardiography parasternal long axis view: hypertrophic cardiomyopathy predominantly septal (IVS-interventricular septum, LPW-left posterior ventricular wall, LA-left atrium, AoV-aortic valve)

**Fig. 3:**
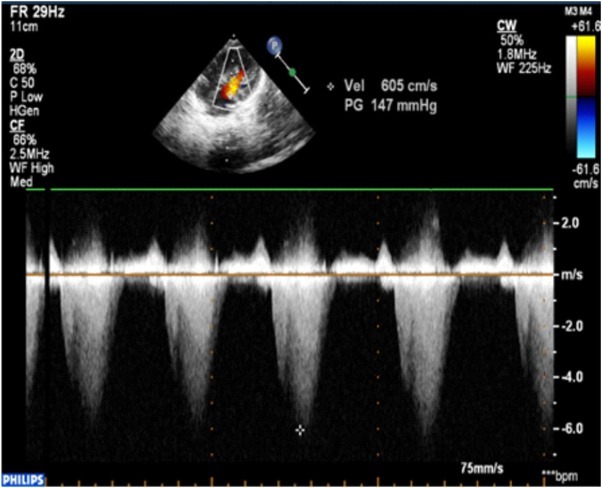
Continuous-wave Doppler apical 4 chambers view: hypertrophic cardiomyopathy predominantly septal with continuous-wave Doppler with a peak gradient in LVOT of 147 mmHg (LVOT-left ventricular outflow tract)

2D, color and continuous-wave Doppler echocardiography showed obstructive hypertrophic cardiomyopathy (OHCM) predominantly in the septal region (Fig. 2, 3), pulmonary infundibular and valvular stenosis as well as a small ASD with a left to right shunt.

### Therapeutic approach and follow-up

Treatment with Propranolol was initiated, however the patient presented functional hemodynamic instability (hypotension, bradycardia, irritability, and psychomotor agitation) which required subsequent dose adjustments.

Due to the suggestive phenotype for NS, molecular genetic testing for RASopathies was performed in a center abroad and revealed the presence of RAF-1 mutation. Due to rapid progression of the gradient in the left ventricular outflow tract (LVOT) and increasing severity of infundibular and valvular pulmonary stenosis with the occurrence of heart failure (growth retardation, fatigue while feeding, tachypnea, respiratory functional syndrome, hepatomegaly), - evolution is shown in Table 1- surgical correction was performed at 14 months of age, consisting of biventricular septal myectomy and transannular patch enlargement of the right ventricular outflow tract and pulmonary artery. In the early postoperative period, the patient developed episodes of sustained ventricular tachycardia with hemodynamic instability, for which treatment with Amiodarone was initiated as well as continuing Propranolol therapy with successful conversion to sinus rhythm. At 3-month follow-up, the patient was hemo-dynamically stable, with a heart rate of 110 beats/minute, in sinus rhythm and gaining weight. Transthoracic echocardiography revealed a residual LVOT mean gradient of 60 mmHg, with preserved biventricular systolic and diastolic function. 24-hour Holter monitoring did not reveal arrhythmias, therefore Amiodarone therapy was ceased, while chronic drug therapy with Propranolol continued.

**Table 1:** Evolutive state of the patient ( IVS (s) – interventicular septum – systolic, IVS (d) – interventricular septum – diastolic, LVOT – left ventricular outflow tract)

Parameters /Age	2 months old	5 months old	8 months old	11 months old
IVS (s)/IVS (d) - cm	2.03	2.18/2.10		2.12/1.85
Peak gradient of Pulmonary stenosis valve (mmHg)	57	75	74	128
Peak gradient of pulmonary infundibular stenosis (mmHg)	28	63	63	100
Peak gradient of LVOT(mmHg)	60	140	95	78
Ponderal index /grade of dystrophy	0.84/ dystrophy grade	0.7/dystrophy grade II	0.62/dystrophy grade III	0.61/dystrophy grade III

## Discussion

NS includes various degrees of clinical manifestations with multiple congenital anomalies, therefore, a scoring system was created by Burgt I et al in 1994, to facilitate the diagnostic process (10). The scoring system includes major and minor criteria regarding facial features, cardiac defects, growth pattern, chest deformity, family history and others. Definitive diagnosis is established by the presence of two major criteria; one major criterion plus two minor criteria; two major criteria and one minor criterion or four minor criteria (10). The scoring system is important for early recognition and specific treatment. However, despite the presence of the scoring system, performing genetic testing, whenever possible, is important (10). The diagnosis of a genetic mutation allows the clinician to predict the severity of cardiac abnormalities, patient’s evolution, as well as overall prognosis according to the cardiovascular disease severity. Therefore, an optimal therapeutic management and complications prevention strategy can be properly achieved. In our case, diagnosis was established through the presence of three major criteria (facial dysmorphism, HCM and PS, grade 1 malnutrition) and the genetic mutation in RAF1 gene of 770C> T. This type of mutation is identified in NS with multiple lentigines as well (formerly named LEOPARD syndrome), with increased prevalence for HCM and frequent right ventricle and right ventricular outflow tract localization, with a worse prognosis compared to other forms (11). HCM is present in 20% of cases with NS due to non-sarcomere genetic mutations, entailing a worse prognosis compared to the idiopathic or familial type (12). Mutations in PTPN 11 gene are described in nearly 50% of cases, while mutations in SOS 1, RAF 1 and KARS genes are described in 25%, 15% and 5% respectively. Other gene mutations like NRAS, BRAF and MAP2K1 were described as well (13). Moreover, the risk of cardiac arrhythmias in patients with RASo-pathies (Costello syndrome, Noonan syndrome and Noonan syndrome with multiple lentigines) is well known due to the presence of cardiac electrophysiological abnormalities. Their presence in the first day of life can predict poor prognosis and a high mortality risk. Danielle t. et al reported a case of two premature infants with clinical features of NS with HCM. One of them presented accelerated ventricular rhythm in the first day of life, which was initially managed with a continuous esmolol drip and ultimately transitioned to propranolol. However, due to worsening of cardiac function, the death of the patient occurred despite aggressive medical management (14). These cardiac arrhythmias, in the absence of antiarrhythmic therapy, can cause hemodynamic instability and the response to antiarrhythmic therapy is particular (14). In the presented case, ECG at presentation showed polymorphic extrasystoles for which treatment with Propranolol was initiated with subsequent dose adjustments due to poor tolerance. John L. Colquitt et. al retrospectively reviewed the medical records of patients with NS who were evaluated in their institution between 1963 and 2011. Their study demonstrated that most patients with NS have stable cardiac diseases with time, and prognosis depends primarily on the severity of the disease at presentation. Severe pathology, typically symptomatic in the form of severe PS and/or severe HCM, usually presents early in life, and these patients are at greatest risk for long-term morbidities (9). In our case, the first presentation was early (2 months old) and due to rapid progression of cardiac anomalies with the occurrence of heart failure, surgical correction was performed at 14 months of age with severe complications. The presence of pre-operative and post-operative cardiac arrhythmia can be a consequence of pressure changes in the context of OHCM. Studies have shown that in patients with increased shortening fraction, intracellular calcium concentration is increased due to the interdependence of intracellular calcium concentration and the force of myocardial contraction (15). In our case, postoperative episodes of ventricular tachycardia with hemodynamic instability were likely due to the existence of a highly arrhythmogenic substrate, with disruption of calcium release from the endoplasmic reticulum on account of the genetic mutation. Taking the abovementioned aspects into consideration, the risk of cardiac arrhythmia recurrence and the gradient recovery in the LVOT should be monitored. The timing, course, and prognosis of NS associated with HCM are variable and not well understood. Therefore, therapeutic approach should be individualized to each patient based on clinical features, results of molecular genetic testing for RASopathies, co-morbidities as well as evolution of cardiac diseases.

## Conclusions

The presented case report describes a case of NS caused by a less common genetic mutation while presenting a rare combination of cardiac malformations. It certifies the importance of recognizing the genetic mutation in patients with NS, which allows predicting the severity of cardiac abnormalities and therefore establishing a proper therapeutic management of these patients.
